# Association between neutrophil-percentage-to-albumin ratio and diabetic kidney disease in type 2 diabetes mellitus patients: a cross-sectional study from NHANES 2009–2018

**DOI:** 10.3389/fendo.2025.1552772

**Published:** 2025-03-06

**Authors:** Hongying Li, Yue Xu, Shuhan Fan, Ziming Wang, Hao Chen, Lin Zhang, Yun Lu, Yifan Miao

**Affiliations:** ^1^ School of Clinical Medicine, Chengdu University of Traditional Chinese Medicine, Chengdu, Sichuan, China; ^2^ Department of Emergency Medicine, Hospital of Chengdu University of Traditional Chinese Medicine, Chengdu, Sichuan, China

**Keywords:** neutrophil-percentage-to-albumin ratio, type 2 diabetes mellitus, National Health and Nutrition Examination Survey (NHANES), inflammation, diabetic kidney disease

## Abstract

**Background:**

The neutrophil-percentage-to-albumin ratio (NPAR), as a low-cost and easily accessible inflammatory biomarker, has garnered considerable attention in various disease studies in recent years. Specifically, existing research has suggested a significant correlation between NPAR and diabetic retinopathy, indicating its potential relevance to diabetic complications. However, despite diabetic kidney disease (DKD) being a complication that severely affects the quality of life of diabetic patients, the association between the prevalence of DKD and NPAR remains to be elucidated. Therefore, this study aims to explore the potential link between NPAR and DKD in patients with type 2 diabetes mellitus.

**Methods:**

We extracted complete data on neutrophil percentage, plasma albumin, serum creatinine, and urine albumin-to-creatinine ratio from the National Health and Nutrition Examination Survey database spanning from 2009 to 2018. Multivariable logistic regression models were employed to examine the relationship between NPAR levels and DKD, and conducted sensitivity tests, subsequently employing Generalized Additive Models combined with smooth curve fitting methods to explore the relationships among variables. Then, subgroup analyses were conducted on the association between NPAR and DKD to investigate changes in the relationship across different subgroups. Finally, Receiver operating characteristic curves were used to assess the predictive performance of the independent variable, NPAR, for the dependent variable, DKD.

**Results:**

A total of 2,263 participants were enrolled in this cross-sectional study. After adjusting for confounding factors, the odds ratio for DKD was 1.44 (95% CI: 1.08-1.90) for the second quartile group, 1.75 (95% CI: 1.33-2.31) for the third quartile group, and 2.95 (95% CI: 2.22-3.93) for the fourth quartile group. Among patients with type 2 diabetes mellitus, a positive correlation was observed between NPAR and DKD. Results from subgroup analyses showed no significant differences among different populations. Receiver operating characteristic (ROC) analysis indicated that NPAR had good predictive performance for DKD.

**Conclusion:**

The prevalence of DKD indicated a positive association with NPAR among individuals with T2DM. Additional large-scale prospective investigations may be helpful in corroborating these findings.

## 1 Introduction

As the most severe and prevalent chronic disease in contemporary society, diabetes mellitus can lead to various life-threatening and costly complications (1). In 2021, more than one in every ten adults globally suffered from diabetes, with China, India, Pakistan, and the United States, among other populous countries, being particularly affected, and the patient population continues to expand rapidly (2). Up to half of diabetic patients develop diabetic kidney disease (DKD), which is a major cause of end-stage kidney disease. Patients with end-stage kidney disease face the threat of cardiovascular diseases (CVD) and rely heavily on costly treatments such as dialysis or kidney transplantation, placing a heavy burden on society and patients’ families. Therefore, early identification and intervention in DKD are crucial for delaying disease progression, reducing complications, and improving prognosis (3, 4).

The pathological process of DKD involves multiple interactions including hyperglycemia, inflammation, lipid accumulation, oxidative stress, renin-angiotensin-aldosterone system, endoplasmic reticulum stress and so on. The primary pathogenesis is related to metabolic disturbances, hemodynamic abnormalities, and chronic inflammatory responses. In-depth research into the pathological mechanisms of DKD has also promoted the development of new diagnostic biomarkers (5), thereby increasing diagnostic methods and reducing examination costs. White blood cell count is a simple and economical indicator for assessing inflammatory status. Takahashi et al. proposed that the spontaneous adhesion of neutrophils in patients with type 2 diabetes mellitus (T2DM) is likely positively correlated with albuminuria (6), and polymorphonuclear neutrophil activation can stem from metabolic disturbances (7). Additionally, the clinical state of chronic inflammation often leads to hypoalbuminemia, and studies have demonstrated that albumin detection indices can serve as biomarkers for estimating the severity of DKD (8). Recently, studies combining these two markers have found that the neutrophil-percentage-to-albumin ratio (NPAR) can be used as a diagnostic indicator for inflammatory-based conditions such as heart failure, septic shock, non-alcoholic fatty liver disease and fibrosis, and tuberculosis (9–12). Furthermore, NPAR has been associated with increased risk of mortality in patients with acute kidney injury and an elevated risk of chronic kidney disease (CKD), and it has shown potential in predicting the duration of hospitalization for T2DM and the occurrence of diabetic retinopathy (13–16).

However, to our knowledge, the correlation between NPAR and DKD has not been previously discussed. Therefore, this study seeks to explore the potential correlation between NPAR and the prevalence of DKD using the National Health and Nutrition Examination Survey (NHANES) database, with the hope of contributing new insights to the understanding of DKD.

## 2 Materials and methods

### 2.1 Selection of study population

**Figure 1:**
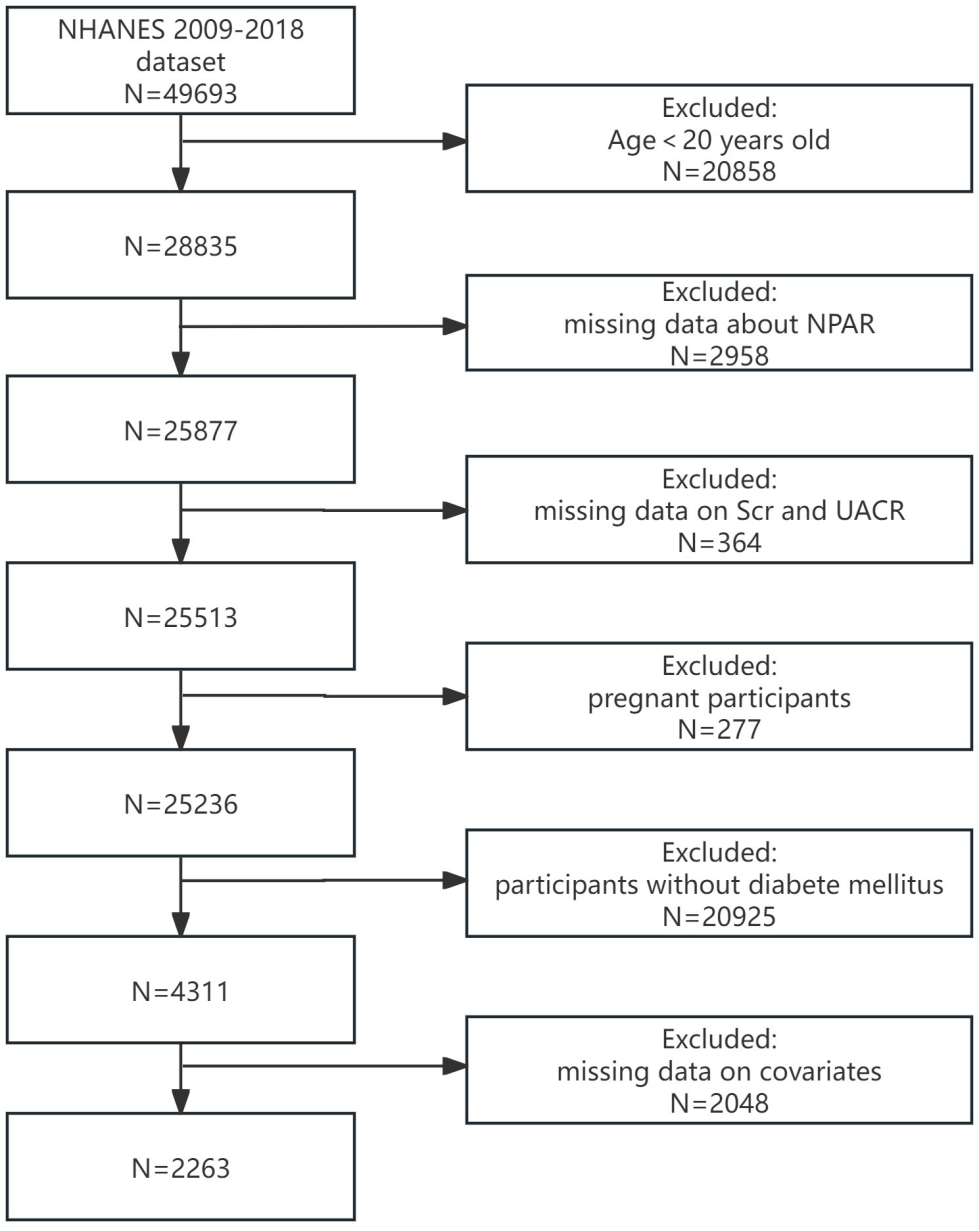
Flowchart of the sample selection.

In this study, all data utilized were extracted from the NHANES, renowned for its national representativeness, comprehensiveness, long-term stability, and reliability. This database encompasses not only basic biological indicators, lifestyles, and anthropometric measurements but also delves into the prevalence of chronic diseases such as diabetes, CVD, and assessments of other health issues, rendering it highly valuable for medical research. These data can be accessed via https://www.cdc.gov/nchs/nhanes. The relevant procedures for obtaining these data have been formally approved by the Ethics Review Committee of the National Center for Health Statistics, and all participants have provided informed consent after being fully informed. We selected the NHANES data from 2009 to 2018 to evaluate the relationship between NPAR and DKD among diabetic patients. After excluding individuals younger than 20 years old (n=20,858), those with incomplete data on neutrophil percentage and plasma albumin (n=2,958), serum creatinine (Scr) and urine albumin-to-creatinine ratio (ACR) (n=364). Furthermore, we excluded pregnant individuals (n=277), those without diabetes (n=20,925), and individuals with missing information on other covariates (n=2,048). Ultimately, 2,263 participants were included in the final analysis (**Figure 1**).

### 2.2 Definition of T2DM

Based on previous literature, the diagnosis can be made after meeting any of the following criteria (17): (1)Self-reported diagnosis by a physician; (2) Current use of hypoglycemic medications or insulin injections; (3) Random blood glucose level ≥ 11.1 mmol/L; (4) Glycated hemoglobin level ≥ 6.5%; (5) Fasting plasma glucose level ≥ 7.0 mmol/L; (6) 2-hour post-load plasma glucose level during an oral glucose tolerance test ≥ 11.1 mmol/L.

### 2.3 Definition of NPAR

Blood samples provided by the subjects are prepared and analyzed by professionally trained laboratory personnel. The percentage of neutrophils is determined using the VCS technology of the Beckman Coulter instrument, in conjunction with an automatic diluting and mixing device and a single-beam photometer, for the classification and enumeration of blood cells. Additionally, in the same sample, the bromocresol purple dye method is employed to detect albumin by its specific binding and color change within a pH range of 5.2-6.8. The albumin concentration is measured through a two-point endpoint reaction by assessing the absorbance at 600 nm (with a reference wavelength of 700 nm). Detailed and comprehensive descriptions of the laboratory methods are accessible on the NHANES website. Subsequently, the NPAR is calculated using the following formula: neutrophil percentage (%)/albumin (g/dL).

### 2.4 Definition of DKD

Scr is converted into creatine, sarcosine, and ultimately hydrogen peroxide through an enzymatic cascade reaction. The hydrogen peroxide, in the presence of peroxidase and a chromogenic substrate, produces a colored product, and its absorbance at 546 nm (with a reference wavelength of 700 nm) is measured to determine Scr levels. For urine protein measurement, a solid-phase fluorescence immunoassay is used, where the fluorescence intensity reflects the amount of urine albumin. Urine creatinine is determined using an enzymatic method, which involves converting creatinine into a measurable colored product through an enzymatic reaction, with the color intensity being directly proportional to the measured value at a wavelength of 546 nm. Subsequently, the estimated glomerular filtration rate (eGFR) is calculated using the Chronic Kidney Disease Epidemiology Collaboration equation, which is applicable to different races and populations. Meanwhile, the official documentation elucidates that the variable ACR has been established using the following formula: URDACT=URXUMA/URXUCR x 100, round to.01. Based on previous studies, the diagnosis of DKD in patients with T2DM was based on the presence of an eGFR < 60 mL/min/1.73m² and/or an ACR ≥ 30 mg/g.

### 2.5 Covariates

The covariates in this study were selected based on factors previously reported to be associated with NPAR and DKD, including: age, gender, race, marital status, education level, smoking status, body mass index (BMI) (<25/<30/≥30 kg/m²), CVD, hypertension (HBP), aspartate aminotransferase (AST), alanine aminotransferase (ALT), triglycerides, low-density lipoprotein (LDL), high-density lipoprotein (HDL), and total cholesterol (TC).

CVD was defined as the presence of at least one of the following conditions: congestive heart failure, coronary heart disease, angina pectoris, myocardial infarction, or stroke. Additionally, HBP was defined as meeting any of the following criteria: (1) average systolic blood pressure ≥ 140 mmHg; (2) average diastolic blood pressure ≥ 90 mmHg; (3) self-reported diagnosis of HBP; (4) current use of antihypertensive medications (18).

### 2.6 Statistical analysis

Given the data collection characteristics of the NHANES database, continuous variables were presented as weighted means with their standard errors, while categorical variables were presented through unweighted counts and weighted proportions. Baseline characteristics across different NPAR quartiles were assessed using the weighted linear regression model and weighted Chi-square test. Multivariable logistic regression analysis was employed to evaluate the association between NPAR and DKD in diabetic patients across different models. Model 1: a crude model including only the neutrophil-to-albumin ratio. Model 2: adjusted for the covariates of age, gender, and race. Model 3: further adjusted for marital status, education level, smoking status, BMI, CVD, HBP, AST, ALT, triglycerides, LDL, HDL, and TC. Sensitivity analyses were conducted by categorizing NPAR into quartiles to verify the robustness of the conclusions. To address the non-linear association observed between NPAR and DKD, generalized additive models with smooth curve fitting techniques were utilized. Subsequently, the population was stratified based on various factors including age (<60/≥60 years), gender (male/female), race (Black/other), BMI (<25/<30/≥30 kg/m²), HBP (yes/no), and CVD (yes/no), to investigate whether the outcome is affected across different strata of the population. Additionally, receiver operating characteristic (ROC) analysis was used to assess the predictive performance of the predictor variable NPAR for the outcome variable DKD. In the statistical analysis of our study, to determine whether the statistical differences were significant, we established a criterion by adopting a two-tailed test and setting the threshold for the p-value to be less than 0.05. For data processing, we selected the Empower software toolkit (http://www.empowerstats.com) in conjunction with R language version 4.1.3 (https://cran.r-project.org/bin/windows/base/old/4.1.3/) to execute all data analyses.

## 3 Results

### 3.1 Baseline characteristics of participants

This study involved 2,263 participants with a mean age of 59.41 ± 13.27 years and 53.76% were male. Based on low-eGFR and albuminuria, 878 (35.47%) participants were diagnosed with DKD. The prevalences of low-eGFR and albuminuria were found to be 410 (16.49%) and 671 (26.29%). Besides, the mean NPAR value was 1.13 ± 0.46. Across different quantile intervals of the NPAR index, it was observed that as the quantile increased (from lower to higher quantiles), and corresponding to the NPAR values within each interval, the number of patients with DKD, low-eGFR, and albuminuria gradually increased. Furthermore, as shown in **Table 1**, significant statistical differences were found in the demographic and clinical characteristics of the study population, including age, gender, race, education level, marital status, BMI, smoking status, HDL-C, triglycerides, ALT, AST, ACR, eGFR, DKD, and albuminuria.

**Table 1:** Baseline characteristics of included participants (n=2263) in the NHANES 2009-2018.

Variable	Overall	Q1	Q2	Q3	Q4	P-value
	n=2263 1.13 ± 0.46	n=564 0.62 ± 0.11	n=532 0.89 ± 0.06	n=598 1.13 ± 0.08	n=569 1.70 ± 0.41	
Age (years)	59.41 ± 13.27	60.02 ± 12.20	60.47 ± 13.73	58.80 ± 13.12	58.71 ± 13.73	0.0767
Gender (%)						<0.0001
Male	1223 (53.76)	320 (56.2)	311 (60.39)	312 (53.27)	280 (47.05)	
Female	1040 (46.24)	244 (43.8)	221 (39.61)	286 (46.73)	289 (52.95)	
Race (%)						<0.0001
Mexican American	399 (10.45)	90 (10.09)	102 (11.36)	116 (10.74)	91 (9.68)	
Other Hispanic	269 (6.35)	54 (6.06)	70 (6.48)	73 (6.1)	72 (6.72)	
Non-Hispanic white	760 (59.85)	128 (47.51)	163 (58.39)	213 (62.6)	256 (67.5)	
Non-Hispanic black	537 (14.06)	203 (24.62)	121 (13.76)	127 (12.23)	86 (8.26)	
Others	298 (9.29)	89 (11.71)	76 (10.02)	69 (8.34)	64 (7.84)	
Education level (%)						0.0386
Less than high school	742 (22.47)	185 (24.95)	181 (21.94)	192 (21.3)	184 (22.23)	
High school or GED	513 (24.15)	123 (21.46)	124 (24.04)	138 (26.05)	128 (24.33)	
Above high school	1004 (53.33)	255 (53.52)	226 (53.96)	266 (52.59)	257 (53.43)	
Marital status (%)						0.0003
Married	1273 (58.74)	311 (56.1)	312 (60.69)	339 (59.31)	311 (58.54)	
Widowed	280 (9.9)	67 (7.74)	78 (13.88)	59 (7.63)	76 (10.56)	
Divorced	281 (12.42)	72 (12.9)	62 (11.7)	80 (12.7)	67 (12.37)	
Separated	90 (2.65)	19 (2.4)	18 (2.16)	33 (3.45)	20 (2.42)	
Never married	227 (10.61)	65 (13.91)	41 (7.39)	57 (11.63)	64 (9.73)	
Living with partner	109 (5.48)	30 (6.95)	21 (4.17)	28 (4.53)	30 (6.38)	
BMI (%)						<0.0001
<25	307 (11.27)	106 (17.53)	73 (11.68)	60 (6.38)	68 (11.12)	
<30	647 (26.14)	181 (31.63)	190 (29.6)	154 (25.78)	122 (19.48)	
≥30	1275 (62.6)	273 (50.83)	261 (58.72)	374 (67.84)	367 (69.4)	
Smoking status (%)						<0.0001
≥100 cigarettes lifetime	1110 (49.37)	235 (37.66)	243 (46.84)	302 (52.46)	330 (57.08)	
<100 cigarettes lifetime	1152 (50.59)	328 (62.14)	289 (53.16)	296 (47.54)	239 (42.92)	
HBP (%)	1760 (77.11)	444 (75.33)	413 (78.62)	464 (76.6)	439 (77.73)	0.6244
CVD (%)	752 (33.24)	163 (29.86)	167 (32.78)	210 (33.71)	212 (35.68)	0.2331
HDL-C, mg/dL	47.82 ± 15.27	51.93 ± 14.78	48.08 ± 13.63	47.05 ± 18.98	45.29 ± 11.62	<0.0001
TC, mg/dL	181.94 ± 45.24	184.17 ± 43.60	185.32 ± 44.58	180.25 ± 44.7 1	179.21 ± 47.19	0.0818
Triglyceride, mg/dL	160.04 ± 132.99	137.36 ± 109.94	165.78 ± 151.91	170.03 ± 123.89	162.33 ± 139.07	0.0006
LDL-C, mg/dL	103.40 ± 38.20	105.51 ± 37.31	105.37 ± 37.34	100.52 ± 36.59	103.11 ± 40.84	0.1211
ALT, U/L	27.67 ± 17.85	29.60 ± 19.81	27.97 ± 16.96	28.70 ± 18.87	24.96 ± 15.49	0.0001
AST, U/L	25.82 ± 13.59	28.31 ± 15.47	25.76 ± 10.94	26.52 ± 15.36	23.30 ± 11.55	<0.0001
ACR, mg/g	133.64 ± 636.46	36.39 ± 154.27	71.78 ± 273.61	103.24 ± 429.59	286.99 ± 1069.83	<0.0001
eGFR, mL/min/1.73 m2	85.36 ± 24.44	86.96 ± 22.20	83.75 ± 23.41	86.94 ± 23.92	83.88 ± 27.05	0.0349
DKD (%)	878 (35.47)	157 (23.32)	192 (35.63)	240 (36.26)	289 (43.65)	<0.0001
Albuminuria (%)	671 (26.29)	111(15.61)	140 (22.59)	192 (28.62)	228(34.92)	<0.0001
Low-eGFR (%)	410 (16.49)	72 (12.26)	92 (17.67)	98 (13.91)	148 (21.29)	0.0001
Low-eGFR and Albuminuria (%)	203 (7.31)	26 (4.55)	40 (4.63)	50 (6.27)	87 (12.57)	<0.0001

### 3.2 Association analysis outcomes

As shown in **Table 2**, NPAR appeared to be positively correlated with DKD (OR=2.29, 95% CI: 1.89-2.78). When NPAR was divided into tertiles, DKD prevalence increased with higher NPAR levels. Further analyses across different models maintained significant associations. In fully adjusted Model 3, when NPAR was analyzed as a continuous variable,a 1-unit increase in NPAR was associated with a 1.56-fold increase in DKD prevalence. Additionally, in quartile-stratified NPAR, the ORs for the Q2, Q3, and Q4 groups were 1.44 (95% CI: 1.08-1.90), 1.75 (95% CI: 1.33-2.31), and 2.95 (95% CI: 2.22-3.93).

**Table 2:** The association between NPAR with DKD, low-eGFR and albuminuria.

Models	NPAR (Continuous)	NPAR (As Quartiles)				
	OR (95%CI)	Q1 (Reference)	Q2 Group OR(95%CI)	Q3 Group OR(95%CI)	Q4 Group OR(95%CI)	*P* for Trend
DKD						
Model1	2.29(1.89-2.78)***	1.00	1.46(1.13-1.89)**	1.74(1.36-2.22)***	2.68(2.09-3.42)***	<0.001
Model2	2.70(2.19-3.34)***	1.00	1.45(1.11-1.90)**	1.90(1.46-2.46)***	3.14(2.40-4.09)***	<0.001
Model3	2.56(2.04-3.22)***	1.00	1.44(1.08-1.90)*	1.75(1.33-2.31)***	2.95(2.22-3.93)***	<0.001
low-eGFR						
Model1	1.89(1.53-2.34)***	1.00	1.43(1.02-2.00)*	1.34(0.96-1.86)	2.40(1.76-3.28)***	<0.001
Model2	2.26(1.76-2.91)***	1.00	1.30(0.90-1.88)	1.40(0.98-2.02)	2.82(1.98-4.03)***	<0.001
Model3	1.94(1.48-2.54)***	1.00	1.29(0.87-1.91)	1.21(0.82-1.78)	2.36(1.61-3.46)***	<0.001
Albuminuria						
Model1	2.12(1.74-2.57)***	1.00	1.46(1.10-1.94)**	1.93(1.47-2.53)***	2.73(2.09-3.56)***	<0.001
Model2	2.39(1.95-2.94)***	1.00	1.48(1.11-1.97)**	2.09(1.59-2.75)***	3.14(2.38-4.15)***	<0.001
Model3	2.44(1.95-3.06)***	1.00	1.43(1.06-1.94)*	1.99(1.48-2.67)***	3.13(2.32-4.23)***	<0.001

OR, odds ratio; 95%CI, 95% confidence interval.

*p < 0.05.

**p < 0.01.

***p < 0.001.

Model 1: crude model. Model 2: adjusted for demographic characteristics including age, gender, and race. Model 3: further adjusted for age, gender, race, education, marital status, HDL-C, TC, triglycerides, LDL-C, smoking-status, HBP, CVD, BMI, ALT, and AST.

Similarly, the study suggested that the group with low-eGFR and albuminuria may also have a positive correlation with the prevalence of DKD. Notably, these relationships persisted even in Model 3 after adjusting for multiple covariates. The odds ratios for continuous variable NPAR with the low-eGFR and albuminuria group were (OR=1.94, 95% CI: 1.48-2.54) and (OR=2.44, 95% CI: 1.95-3.06), respectively. In the Q4 group of NPAR quartiles, the corresponding effect sizes were OR=2.36 (95% CI: 1.61-3.46) and OR=3.13 (95% CI: 2.32-4.23).

**Figure 2:**
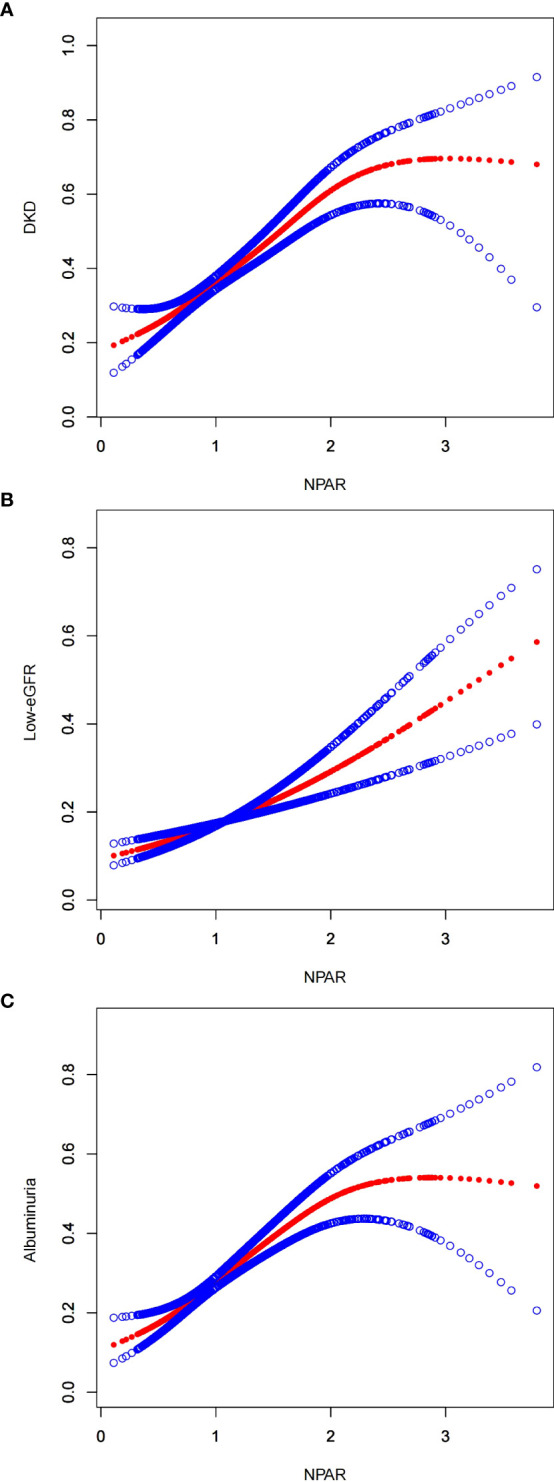
Smooth curve fitting for NPAR with DKD, low-eGFR and albuminuria. **(A)** NPAR and DKD; **(B)** NPAR and low-eGFR; **(C)** NPAR and albuminuria.

Furthermore, this study conducted an in-depth analysis of the associations between NPAR and various subgroups to explore the non-linear relationships between them (**Figure 2**). In the figure, the red line represents the curve fitting, while the blue lines indicate the confidence intervals. Using recursive algorithms, it was suggested that the relationship between NPAR and DKD (p=0.081) as well as the low-eGFR group (p=0.175) does not yet demonstrate a statistically significant non-linear association, whereas a non-linear association was observed with the albuminuria group (p=0.004).

### 3.3 Subgroup analysis

**Figure 3:**
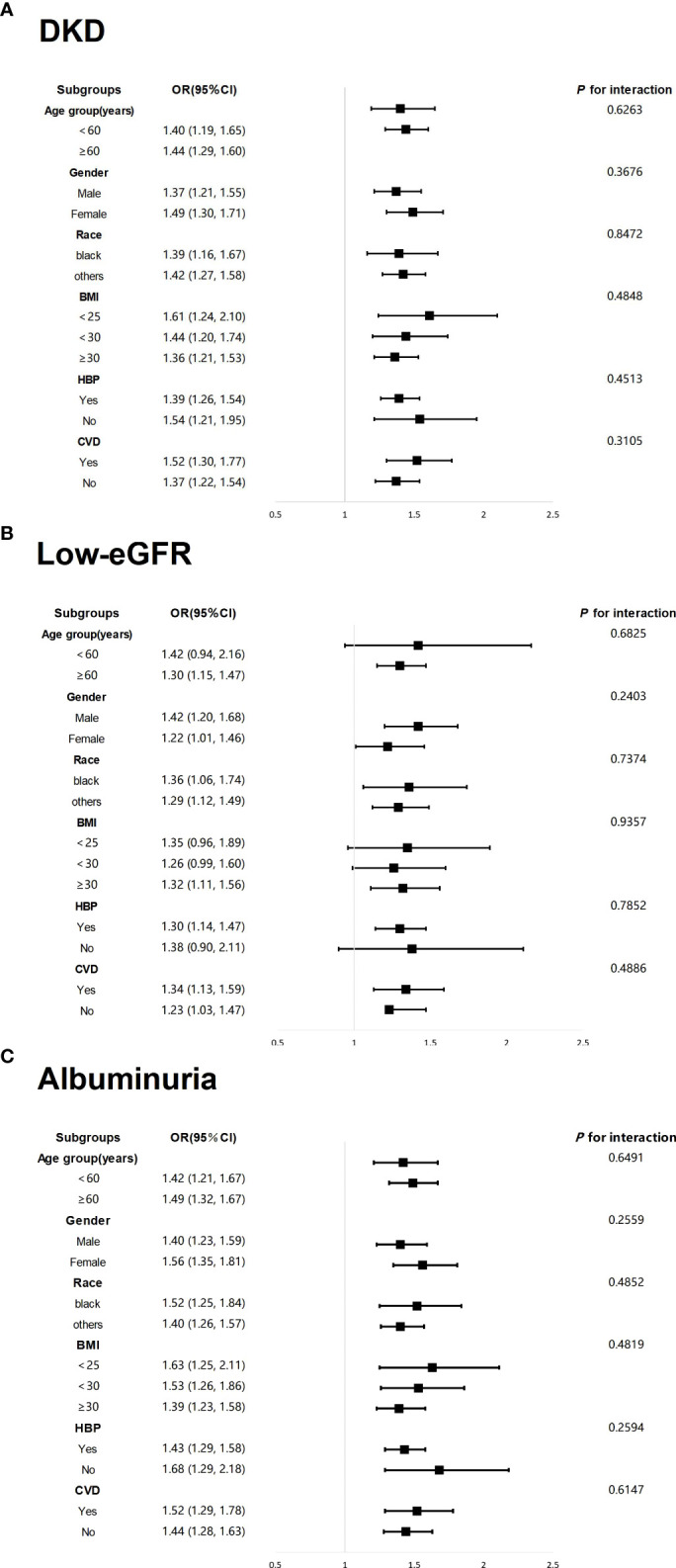
Forest Map-Subgroup analysis for the associations of NPAR with DKD, low-eGFR, and albuminuria. **(A)** NPAR and DKD; **(B)** NPAR and low-eGFR; **(C)** NPAR and albuminuria.

In Model 3, which adjusts for all confounding factors, stratified analyses by age, gender, race, BMI, HBP, and CVD were conducted to assess the consistency of the associations between DKD, low-eGFR, and albuminuria with NPAR across different levels within these subgroups. The results, as shown in **Figure 3**, indicated that the prevalence of DKD, low-eGFR, and albuminuria were positively correlated with NPAR across all subgroups, with no significant differences in the relationships observed among different populations.

### 3.4 ROC analysis

**Figure 4:**
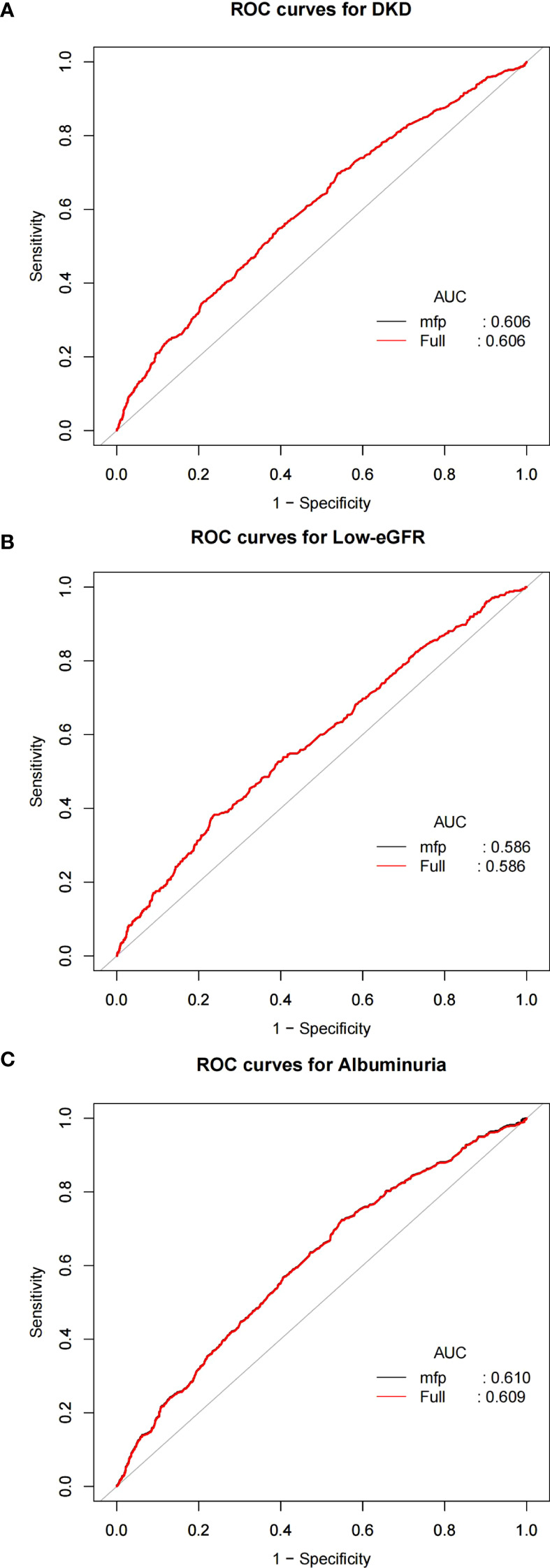
ROC curves and the AUC values of NPAR in diagnosing DKD, low-eGFR and albuminuria. **(A)** NPAR and DKD; **(B)** NPAR and low-eGFR; **(C)** NPAR and albuminuria.

As illustrated in **Figure 4**, to evaluate the predictive ability of NPAR for DKD, low-eGFR, and albuminuria, in the study we calculated the Area Under the Curve values. The relevant findings are presented in **Table 3**. The Area Under the Curve values for NPAR in predicting DKD and albuminuria are higher compared to those for low-eGFR. This indicates that NPAR demonstrates better accuracy and discriminative power in the DKD and albuminuria groups.

**Table 3:** AUC values of NPAR on DKD, low-eGFR and albuminuria.

Test	AUC	95%CI low	95%CI upp	Best threshold	Specificity	Sensitivity
DKD	0.6061	0.5823	0.6299	0.3533	0.4614	0.697
low-eGFR	0.5857	0.555	0.6164	0.195	0.762	0.3829
Albuminuria	0.6088	0.5835	0.634	0.2671	0.4516	0.7228

## 4 Discussion

In this cross-sectional study of 2,263 US adult T2DM patients, we observed a positive correlation between NPAR and the prevalences of DKD, albuminuria, and low-eGFR. This correlation remained stable in subsequent sensitivity tests and subgroup analyses. Finally, ROC analysis suggested that NPAR has certain predictive efficacy for DKD. These findings indicate that NPAR has the potential to become a new biomarker in the clinical diagnosis of DKD.

Our results are in line with those of J. Li et al (14), who found that diabetes significantly affects the NPAR-CKD relationship in diabetic subgroups. We further investigated this link between NPAR and DKD in diabetic patients. However, our age-stratified subgroup analysis did not find any significant effects, which may be related to the characteristics of our study population. The pathogenesis of DKD, a complication of diabetes, differs from that of primary CKD, which is characterized by diverse etiologies and pathological types (19). For example, elderly CKD patients are more prone to HBP and CVD, which can affect the NPAR-CKD relationship (20–22). In our study, we controlled for various confounders and found that the NPAR-DKD association is independent of HBP, CVD, BMI, and other factors. This further supports the potential of NPAR as a valuable diagnostic marker for DKD.

Interestingly, two phenomena that merit attention were observed in our research. Firstly, the analysis showed that ACR has a significantly higher effect size and greater statistical significance in its relationship with NPAR compared to eGFR, indicating a stronger link between ACR and NPAR. ACR reflects the integrity of the glomerular filtration barrier, while eGFR indicates overall filtration function. DKD typically progresses from increased albuminuria to significant albuminuria, followed by a rapid decline in renal function (23). In the early stages of DKD, eGFR may remain stable due to factors such as glomerular hyperfiltration and compensatory mechanisms (24), suggesting that NPAR may serve as a sensitive early warning indicator for DKD. Secondly, in the baseline characteristics table of the participants included, a paradoxical trend was observed between eGFR and low-eGFR with increasing NPAR. To further investigate this relationship, we conducted additional analyses examining the association between eGFR and NPAR, as shown in **Supplementary Table S1**, **Supplementary Figures S1**, **S2**. Significant differences were observed across subgroups stratified by age, HBP, and albuminuria (**Supplementary Figure S2**), indicating that these factors influence the eGFR-NPAR relationship.

Some studies have partially unveiled the potential pathological mechanisms underlying the relationship between NPAR and DKD. Diabetes, characterized by persistent hyperglycemia (25), leads to increased expression of chemokines (e.g., C-C motif chemokine 2) and adhesion molecules (e.g., intercellular adhesion molecule 1), which promote leukocyte migration (e.g., monocytes, neutrophils, lymphocytes) to the kidneys (26), initiating inflammation. This inflammation activates pro-inflammatory pathways in glomerular endothelial cells and podocytes, such as NF-κB, accelerating albumin leakage, raising ACR levels (27) and reducing eGFR (28). Concurrently, Inflammation also reduces insulin sensitivity, exacerbating hyperglycemia and further promoting inflammatory responses (29), creating a vicious cycle of renal damage, tubulointerstitial injury, fibrosis, and worsening kidney function (30). Additionally, as a complex metabolic disease, DKD involves not only inflammation but also classic pathological features of DKD, such as persistent albuminuria and Low-GFR (31), which often lead to decreased serum albumin levels (32). A prospective study showed that elevated inflammatory parameters were independently associated with hypoalbuminemia (33), and hypoalbuminemia can further increase the reabsorptive burden on the renal tubules, thereby exacerbating the decline in eGFR (34).

In recent years, the relationship between leukocyte subtypes and DKD has garnered increasing attention. Various indicators have been explored as economical and accessible clinical risk indicators for DKD (35–38). Compared with previous indicators, NPAR is less affected by acute fluctuations in albumin levels (39), indicating its relative stability in assessing disease risk. Moreover, the combination of NPAR with inflammatory markers and renal function indicators can provide a more comprehensive assessment of the patient’s inflammatory status and kidney function. This integrated approach helps enhance the diagnostic accuracy of DKD.

The data for this study were obtained from the NHANES database, a representative nationwide health and nutrition survey project led and implemented by the Centers for Disease Control and Prevention in the United States. In our study, we considered numerous confounding factors and employed methods such as subgroup analysis and ROC analysis to ensure reliable association results. However, although many important confounding factors were included, the interference of other unmeasured confounding factors cannot be determined conclusively. Additionally, the cross-sectional design of this study limits the ability to establish a causal relationship between NPAR and DKD, allowing only an assessment of their association. Therefore, prospective studies are required to clarify the potential causal link and confirm these findings.

## 5 Conclusion

Our research suggests a positive link between NPAR levels among individuals with T2DM and the prevalence of DKD. This relationship appeared to be stable irrespective of differences in gender, age, body mass index, HBP, etc. Nonetheless, additional studies are warranted to substantiate our observations.

## Data Availability

The original contributions presented in the study are included in the article/**Supplementary Material**. Further inquiries can be directed to the corresponding authors.

## References

[1] Heald AH Stedman M Davies M Livingston M Alshames R Lunt M. Estimating life years lost to diabetes: outcomes from analysis of National Diabetes Audit and Office of National Statistics data. Cardiovasc Endocrinol Metab. (2020) 9:183–5. doi: 10.1097/XCE.0000000000000210 PMC7673790 33225235

[2] Sun H Saeedi P Karuranga S Pinkepank M Ogurtsova K Duncan BB. IDF Diabetes Atlas: Global, regional and country-level diabetes prevalence estimates for 2021 and projections for 2045. Diabetes Res Clin Pract. (2022) 183:109119. doi: 10.1016/j.diabres.2021.109119 34879977 PMC11057359

[3] Selby NM Taal MW. An updated overview of diabetic nephropathy: Diagnosis, prognosis, treatment goals and latest guidelines. Diabetes Obes Metab. (2020) 22:3–15. doi: 10.1111/dom.14007 32267079

[4] Zou Y Zhao L Zhang J Wang Y Wu Y Ren H. Development and internal validation of machine learning algorithms for end-stage renal disease risk prediction model of people with type 2 diabetes mellitus and diabetic kidney disease. Ren Fai. (2022) 44:562–70. doi: 10.1080/0886022X.2022.2056053 PMC8986220 35373711

[5] Wang Y Jin M Cheng CK Li Q. Tubular injury in diabetic kidney disease: molecular mechanisms and potential therapeutic perspectives. Front Endocrinol. (2023) 14:1238927. doi: 10.3389/fendo.2023.1238927 PMC10433744 37600689

[6] Takahashi T Hato F Yamane T Inaba M Okuno Y Nishizawa Y. Increased spontaneous adherence of neutrophils from type 2 diabetic patients with overt proteinuria: possible role of the progression of diabetic nephropathy. Diabetes Care. (2000) 23:417–8. doi: 10.2337/diacare.23.3.417 10868876

[7] Wierusz-Wysocka B Wykretowicz A Byks H Sadurska K Wysocki H. Polymorphonuclear neutrophils adherence, superoxide anion. (O2–) production and HBA1 level in diabetic patients. Diabetes Res Clin Pract. (1993) 21:109–14. doi: 10.1016/0168-8227(93)90057-C 8269811

[8] Cai YW Zhang HF Gao JW Cai ZX Cai JW Gao QY. Serum albumin and risk of incident diabetes and diabetic microvascular complications in the UK Biobank cohort. Diabetes Metab. (2023) 49:101472. doi: 10.1016/j.diabet.2023.101472 37678759

[9] Hu Z Wang J Xue Y Zhang Q Xu Q Ji K. The neutrophil-to-albumin ratio as a new predictor of all-cause mortality in patients with heart failure. J Inflammation Res. (2022) 15:701–13. doi: 10.2147/JIR.S349996 PMC8818978 35140500

[10] Gong Y Li D Cheng B Ying B Wang B. Increased neutrophil percentage-to-albumin ratio is associated with all-cause mortality in patients with severe sepsis or septic shock. Epidemiol Infect. (2020) 148:e87. doi: 10.1017/S0950268820000771 32238212 PMC7189348

[11] Bao B Xu S Sun P Zheng L. Neutrophil to albumin ratio: a biomarker in non-alcoholic fatty liver disease and with liver fibrosis. Front Nutr. (2024) 11:1368459. doi: 10.3389/fnut.2024.1368459 38650638 PMC11033504

[12] Stefanescu S Cocoş R Turcu-Stiolica A Shelby ES Matei M Subtirelu MS. Prediction of treatment outcome with inflammatory biomarkers after 2 months of therapy in pulmonary tuberculosis patients: preliminary results. Pathogens. (2021) 10:789. doi: 10.3390/pathogens10070789 34206598 PMC8308673

[13] Wang B Li D Cheng B Ying B Gong Y. The neutrophil percentage-to-albumin ratio is associated with all-cause mortality in critically ill patients with acute kidney injury. BioMed Res Int. (2020) 2020:1–9. doi: 10.1155/2020/5687672 PMC7049452 32219136

[14] Li J Xiang T Chen X Fu P. Neutrophil-percentage-to-albumin ratio is associated with chronic kidney disease: Evidence from NHANES 2009–2018. PloS One. (2024) 19:e0307466. doi: 10.1371/journal.pone.0307466 39102412 PMC11299806

[15] Tan J Zhang Z He Y Yu Y Zheng J Liu Y. A novel model for predicting prolonged stay of patients with type-2 diabetes mellitus: a 13-year. (2010–2022) multicenter retrospective case–control study. J Transl Med. (2023) 21:91. doi: 10.1186/s12967-023-03959-1 36750951 PMC9903472

[16] He X Dai F Zhang X Pan J. The neutrophil percentage-to-albumin ratio is related to the occurrence of diabetic retinopathy. J Clin Lab Anal. (2022) 36:e24334. doi: 10.1002/jcla.24334 35285099 PMC8993596

[17] Zhou H Li T Li J Zhuang X Yang J. The association between visceral adiposity index and risk of type 2 diabetes mellitus. Sci Rep. (2024) 14:16634. doi: 10.1038/s41598-024-67430-x 39025982 PMC11258278

[18] Jaeger BC Sakhuja S Hardy ST Akinyelure OP Bundy JD Muntner P. Predicted cardiovascular risk for United States adults with diabetes, chronic kidney disease, and at least 65 years of age. J Hypertens. (2022) 40:94–101. doi: 10.1097/HJH.0000000000002982 34420013 PMC9259065

[19] Francis A Harhay MN Ong ACM Tummalapalli SL Ortiz A Fogo AB. Chronic kidney disease and the global public health agenda: an international consensus. Nat Rev Nephrol. (2024) 20:473–85. doi: 10.1038/s41581-024-00820-6 38570631

[20] Zhao M Huang X Zhang Y Wang Z Zhang S Peng J. Predictive value of the neutrophil percentage-to-albumin ratio for coronary atherosclerosis severity in patients with CKD. BMC Cardiovasc Disord. (2024) 24:277. doi: 10.1186/s12872-024-03896-x 38807036 PMC11134736

[21] Liu Z Dong L Shen G Sun Y Liu Y Mei J. Associations of neutrophil-percentage-to-albumin ratio level with all-cause mortality and cardiovascular disease-cause mortality among patients with hypertension: evidence from NHANES 1999-2010. Front Cardiovasc Med. (2024) 11:1397422. doi: 10.3389/fcvm.2024.1397422 39087072 PMC11288876

[22] Kurkiewicz K Gąsior M Szyguła-Jurkiewicz BE. Markers of malnutrition, inflammation, and tissue remodeling are associated with 1-year outcomes in patients with advanced heart failure. Pol Arch Intern Med. (2023) 133:16411. doi: 10.20452/pamw.16411 36633195

[23] Yokoyama H Araki S Honjo J Okizaki S Yamada D Shudo R. Association between remission of macroalbuminuria and preservation of renal function in patients with type 2 diabetes with overt proteinuria. Diabetes Care. (2013) 36:3227–33. doi: 10.2337/dc13-0281 PMC3781501 23780946

[24] Kanbay M Copur S Bakir CN Covic A Ortiz A Tuttle KR. Glomerular hyperfiltration as a therapeutic target for CKD. Nephrol Dial Transplant. (2024) 39:1228–38. doi: 10.1093/ndt/gfae027 PMC12086678 38308513

[25] Efiong EE Bazireh H Fuchs M Amadi PU Effa E Sharma S. Crosstalk of hyperglycaemia and cellular mechanisms in the pathogenesis of diabetic kidney disease. Int J Mol Sci. (2024) 25:10882. doi: 10.3390/ijms252010882 39456664 PMC11507194

[26] Navarro-González JF Mora-Fernández C de Fuentes MM García-Pérez J. Inflammatory molecules and pathways in the pathogenesis of diabetic nephropathy. Nat Rev Nephrol. (2011) 7:327–40. doi: 10.1038/nrneph.2011.51 21537349

[27] Hu S Hang X Wei Y Wang H Zhang L Zhao L. Crosstalk among podocytes, glomerular endothelial cells and mesangial cells in diabetic kidney disease: an updated review. Cell Commun Signal CCS. (2024) 22:136. doi: 10.1186/s12964-024-01502-3 38374141 PMC10875896

[28] Thomas HY Ford Versypt AN. Pathophysiology of mesangial expansion in diabetic nephropathy: mesangial structure, glomerular biomechanics, and biochemical signaling and regulation. J Biol Eng. (2022) 16:19. doi: 10.1186/s13036-022-00299-4 35918708 PMC9347079

[29] Weinberg Sibony R Segev O Dor S Raz I. Overview of oxidative stress and inflammation in diabetes. J Diabetes. (2024) 16:e70014. doi: 10.1111/1753-0407.70014 39435991 PMC11494684

[30] Zheng Z Zheng F. Immune cells and inflammation in diabetic nephropathy. J Diabetes Res. (2015) 2016:1841690. doi: 10.1155/2016/1841690 26824038 PMC4707326

[31] Oshima M Shimizu M Yamanouchi M Toyama T Hara A Furuichi K. Trajectories of kidney function in diabetes: a clinicopathological update. Nat Rev Nephrol. (2021) 17:740–50. doi: 10.1038/s41581-021-00462-y 34363037

[32] Wagner M Alam A Zimmermann J Rauh K Koljaja-Batzner A Raff U. Endogenous erythropoietin and the association with inflammation and mortality in diabetic chronic kidney disease. Clin J Am Soc Nephrol CJASN. (2011) 6:1573–9. doi: 10.2215/CJN.00380111 21734083

[33] Eckart A Struja T Kutz A Baumgartner A Baumgartner T Zurfluh S. Relationship of nutritional status, inflammation, and serum albumin levels during acute illness: A prospective study. Am J Med. (2020) 133:713–722.e7. doi: 10.1016/j.amjmed.2019.10.031 31751531

[34] Fogo AB Harris RC. Crosstalk between glomeruli and tubules. Nat Rev Nephrol. (2025) 21:189–99. doi: 10.1038/s41581-024-00907-0 39643696

[35] Cardoso CRL Leite NC Salles GF. Importance of hematological parameters for micro- and macrovascular outcomes in patients with type 2 diabetes: the Rio de Janeiro type 2 diabetes cohort study. Cardiovasc Diabetol. (2021) 20:133. doi: 10.1186/s12933-021-01324-4 34229668 PMC8261940

[36] Fukui M Tanaka M Hamaguchi M Senmaru T Sakabe K Shiraishi E. Eosinophil count is positively correlated with albumin excretion rate in men with type 2 diabetes. Clin J Am Soc Nephrol CJASN. (2009) 4:1761–5. doi: 10.2215/CJN.03330509 PMC2774959 19808222

[37] Li J Wang X Jia W Wang K Wang W Diao W. Association of the systemic immuno-inflammation index, neutrophil-to-lymphocyte ratio, and platelet-to-lymphocyte ratio with diabetic microvascular complications. Front Endocrinol. (2024) 15:1367376. doi: 10.3389/fendo.2024.1367376 PMC11039910 38660516

[38] Tekin BG Pektaş E. Investigation of MHR-nephropathy relationship and the effect of SGLT2is on MHR in patients with type 2 diabetes. Ir J Med Sci. (2024) 193:1283–7. doi: 10.1007/s11845-024-03638-0 38366276

[39] He YS Cao F Musonye HA Xu YQ Gao ZX Ge M. Serum albumin mediates the associations between heavy metals and two novel systemic inflammation indexes among U.S. adults. Ecotoxicol Environ Saf. (2024) 270:115863. doi: 10.1016/j.ecoenv.2023.115863 38134642

