# Supplementary material for: Association between neutrophil-percentage-to-albumin ratio and diabetic kidney disease in type 2 diabetes mellitus patients: a cross-sectional study from NHANES 2009–2018
Source: Front Endocrinol (Lausanne). 2025 Mar 6;16:1552772. doi: 10.3389/fendo.2025.1552772 (PMC11922708; doi:10.3389/fendo.2025.1552772)
Supplement: Supplementary file 1 [file DataSheet1.docx]

Supplementary Material

# Supplementary Table

**TABLE S1 The association between NPAR with eGFR**.

| **Models** | **NPAR (Continuous)** | **NPAR (As Quartiles)** | | | |  |
| --- | --- | --- | --- | --- | --- | --- |
|  | **β (95％CI)** | **Q1 (Reference)** | **Q2 Group β (95％CI)** | **Q3 Group β (95％CI)** | **Q4 Group β (95％CI)** | ***P for Trend*** |
| Model1 | -4.58 (-6.82, -2.35) *** | 0 | -3.80 (-6.77, -0.83) * | -2.14 (-5.03, 0.75) | -6.85 (-9.77, -3.93)*** | <0.001 |
| Model2 | -5.33 (-7.04, -3.62) *** | 0 | -2.54 (-4.77, -0.31) * | -2.53 (-4.70, -0.35) * | -7.31 (-9.55, -5.08)*** | <0.001 |
| Model3 | -4.13 (-5.92, -2.34) *** | 0 | -2.31 (-4.55, -0.07) * | -1.54 (-3.75, 0.66) | -5.84 (-8.16, -3.51)*** | <0.001 |

β: Regression coefficient. 95%CI:95% confidence interval.

* p < 0.05

*** p < 0.001

Model 1: crude model. Model 2: adjusted for demographic characteristics including age, gender, and race. Model 3: further adjusted for age, gender, race, education, marital status, HDL-C, TC, triglycerides, LDL-C, smoking-status, HBP, CVD, BMI, ALT, and AST.

# Supplementary Figures


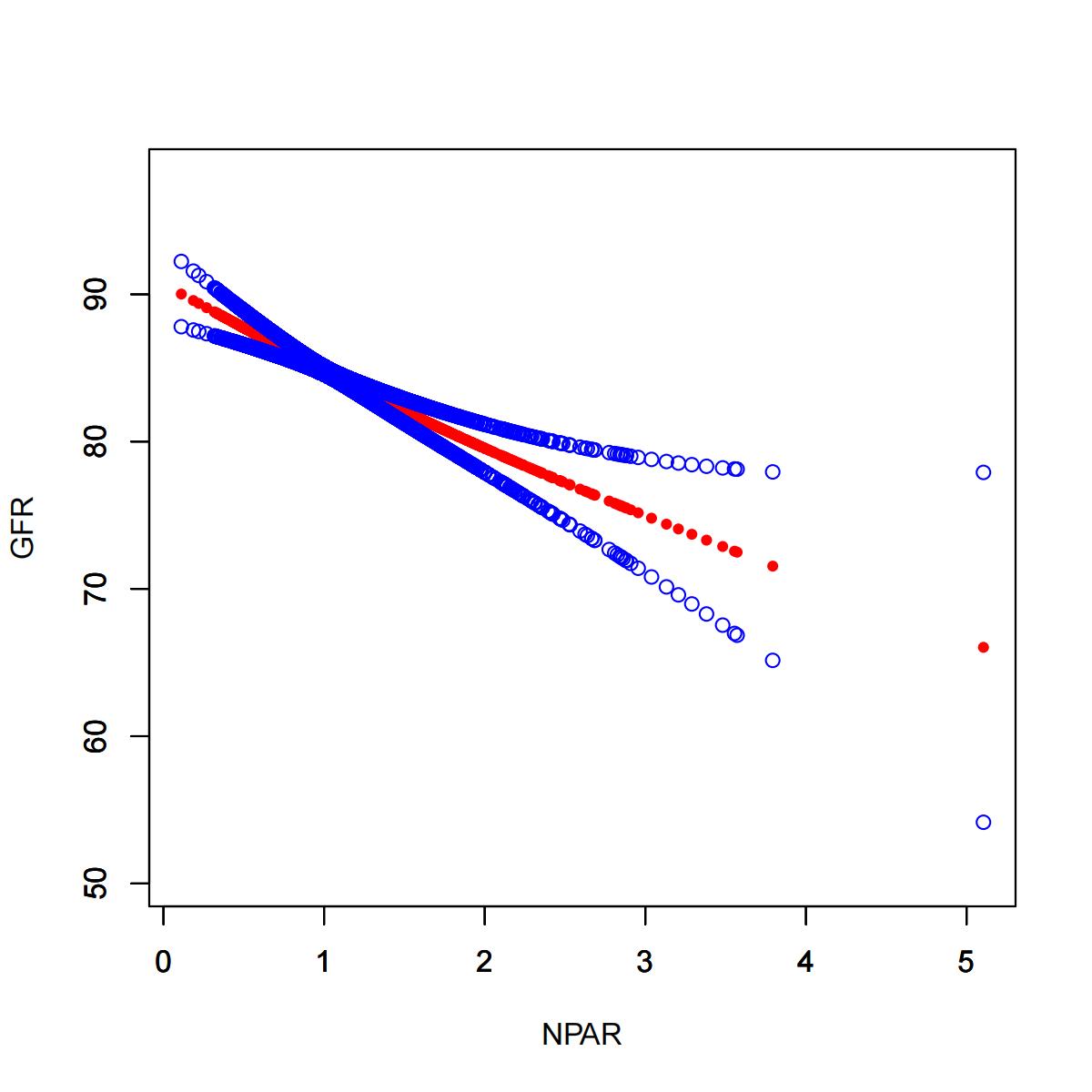


**FIGURE S1** Smooth curve fitting for NPAR with eGFR.


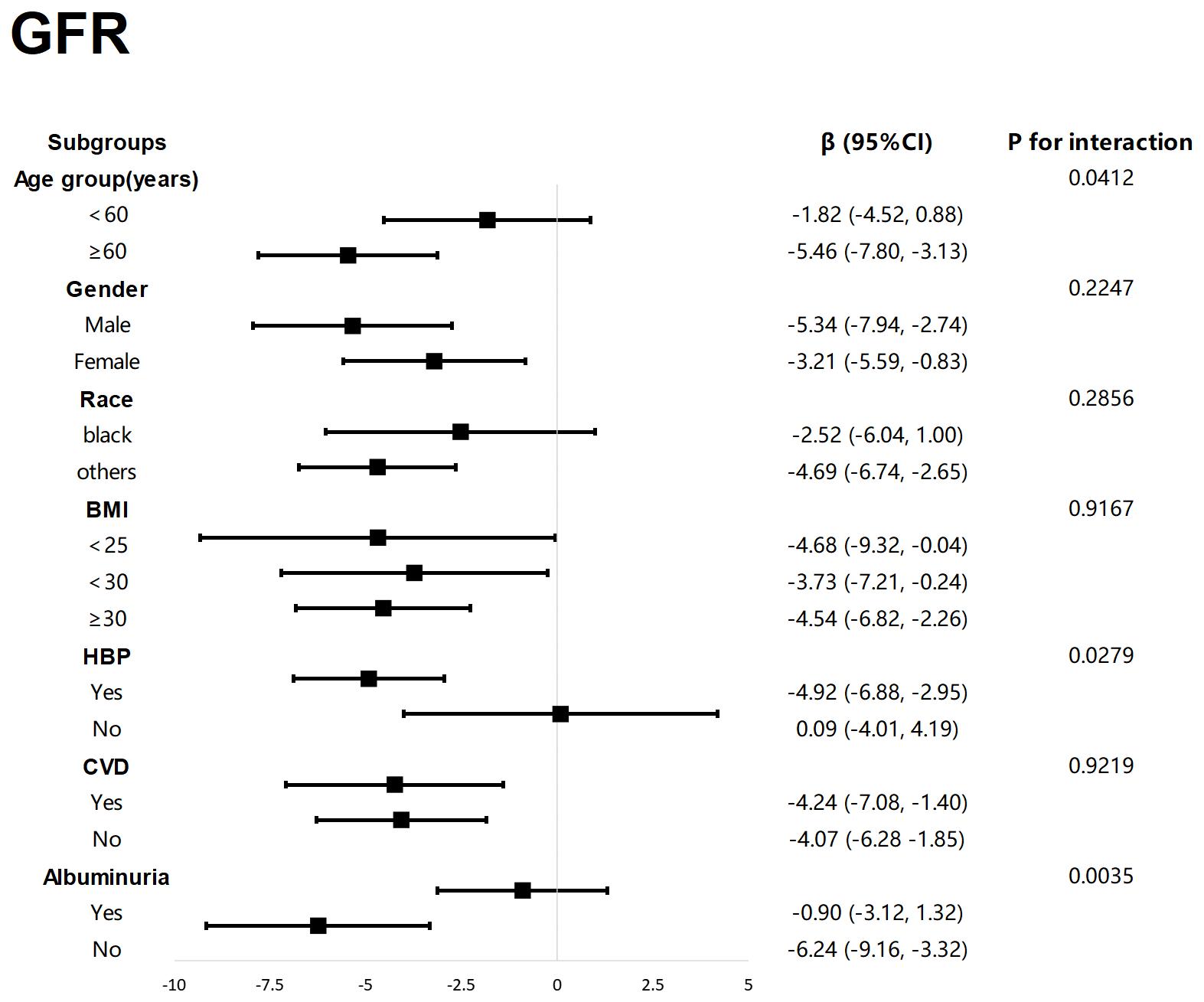


**FIGURE S2** Forest Map-Subgroup analysis for the associations of NPAR with GFR.
